# Incidence of Deep Vein Thrombosis in Hospitalized Chinese Medical Patients and the Impact of DVT Prophylaxis

**DOI:** 10.1155/2011/629383

**Published:** 2011-02-15

**Authors:** Gregory Cheng, Crystal Chan, Ying Ting Liu, Yee Fun Choy, Mei Mei Wong, Pui Kwan Ernest Yeung, Ka Ling Ng, Lai Shan Tsang, Raymond S. M. Wong

**Affiliations:** Department of Medicine and Therapeutics, The Chinese University of Hong Kong, Shatin, Hong Kong

## Abstract

*Objective*. To evaluate the incidence of deep vein thrombosis in hospitalized Chinese medical patients and the impact of DVT prophylaxis. *Methods*. All cases of confirmed proximal DVT from 1 January 2005 to 31 December 2008 were reviewed retrospectively to determine the presence of risk factors and whether DVT developed: during hospitalization in medical wards or in case of readmission with a diagnosis of DVT within 14 days of discharge from a recent admission to medical wards. The impact of prophylaxis will be estimated by comparing the annual incidence of proximal DVT among medical patients hospitalized from 2005 to 2007 with that of 2008 (DVT prophylaxis commonly used). *Results*. From 1 January 2005 to 31 December 2008, 3938 Doppler ultrasound studies were performed for suspected DVT. Proximal DVT was diagnosed in 687 patients. The calculated incidence of proximal DVT among medical patients hospitalized for at least two days was 1.8%, 2%, and 1.7% for the year 2005, 2006, and 2007, respectively. The incidence was 1.1% for 2008 (*P* < .001). *Conclusion*. Proximal DVT was substantial in Chinese medical patients, and DVT prophylaxis might reduce such risk.

## 1. Introduction

Deep venous thrombosis (DVT) and pulmonary embolism (PE) are common and serious complications occurring in hospitalized patients [1, 2]. Many medical conditions such as malignancy, neurological diseases with paresis, cardiac failure, and acute myocardial infarction are associated with increased risk of thromboembolism [3]. Hospitalization for an acute medical illness is independently associated with about an eightfold increased relative risk for VTE [4] and accounts for almost one quarter of all VTE events within the general population [5]. Recently completed trials comparing low-molecular-weight heparins (LMWH) or pentasacharide with placebo controls have provided convincing evidence for VTE prevention in this patient population [6–8]. There was little data about the incidence of DVT among Chinese patients admitted to a medical ward. In the past, it was widely believed that the incidence of DVT was much lower among the Chinese population than Caucasians, and prophylaxis was generally considered not necessary. Recent studies had shown that the incidence of postoperative DVT in Asian countries was significant [9, 10] Asian patients with hip fractures, undergoing total hip and total knee replacements without pharmacological prophylaxis had postoperative DVT rates of 17–58% and proximal DVT rates of 5.8–17.1%. These rates were comparable to that reported in Western populations.. In a retrospective analysis [11], we estimated that the incidence of proximal DVT among Chinese patients admitted to general medical wards was at least three per one thousand patients. Among the medical patients who developed proximal DVT during their hospital stay or within a week of discharge from a medical ward, most of them had risk factors such as malignancy (40%), infections (21%), congestive heart failure (13%), and stroke (7%). In a prospective study [12] using d-dimer screening, the incidence of DVT for Chinese medical patients staying for two or more days was at least 10 per 1000. Among patients with risk factor such as malignancy, stroke heart failure or respiratory failure, the incidence of proximal DVT ranged between 3.8 and 6.3% and was comparable to that reported in Western population. Since 2008, selected patients admitted to medical wards, especially those with acute respiratory failure, congestive heart failure, sepsis underlying malignancy and restricted mobility might be given DVT prophylaxis with low-molecular heparin at physician's discretion. We reported here the incidence of proximal DVT among hospitalized Chinese medical patients in 2008 and compare it with the proximal DVT incidence from 2005–2007, a period when DVT prophylaxis was generally not given. 

## 2. Methods

### 2.1. Study Population

The Prince of Wales Hospital is a general hospital in Hong Kong serving a population of over one million. All Doppler ultrasound studies performed for deep vein thrombosis for the period from 1 January 2005 to 31 December 2008 were reviewed. The medical records of all cases of confirmed proximal DVT were reviewed to determine (a) the presence of risk factors, (b) the number of proximal DVT developed during hospitalization in a medical ward, (c) the number of patients readmitted with proximal DVT within 14 days of discharge from a medical admission of at least two days, (d) whether these patients had received any DVT prophylaxis during the current or previous medical admission.

The total number of admissions to the medical wards during the study period was obtained from the hospital statistics. We excluded short-stay admissions (less than 2 days) for procedures such as biopsy, bronchoscopy, endoscopy, or blood transfusion from analysis. Only medical admissions with a minimum stay of two days and proximal DVT developing during hospitalization in medical wards or readmission with a diagnosis of DVT within 14 days of discharge from a recent admission to medical wards were used to calculate the annual incidence of proximal DVT among medical patients.

### 2.2. Doppler Ultrasound Study

A team of experienced radiologists and qualified sonographers performed the Doppler study using ultrasound scanner (Pulse Echo type Model GELOG10-9). Absent or diminished Doppler flow, lack of respiratory variation, and failure to augment flow with maneuvers (calf compression) were used to establish the diagnosis of proximal and distal DVT.

### 2.3. DVT Prophylaxis for Medical Patients

Patients admitted with a diagnosis of acute respiratory failure, congestive heart failure, sepsis, malignancy, obesity and restricted mobility during hospitalization may be given DVT prophylaxis at the discretion of the attending physicians. Low-molecular weight heparin is the medication most commonly prescribed for this purpose. In our hospital, prophylaxis was usually only given for the hospital stay. It was rarely continued after discharge. Doppler ultrasound will be performed whenever DVT was suspected

### 2.4. Statistical Analysis

The impact of prophylaxis was estimated by comparing the annual incidence of proximal DVT among medical patients hospitalized from 2005 to 2007 with the 2008 annual incidence using chi-square test.

## 3. Results

From 1 January 2005 to 31 December 2008, 3938 Doppler ultrasound studies were performed for suspected DVT. Proximal DVT was diagnosed in 687 patients. Of these, 434(63%) patients developed DVT during hospitalization. DVT developed in these patients after at least 3 days of hospital study (median 8 days, range 3–86 days). Two hundred and fifty-three (37%) subjects were admitted because of DVT developing within 14 days (range 2–14 days, median 6 days) of discharge from a previous medical admission. Median in-hospital stay for the previous medical admissions was 13 days (range 2–166 days). Seventeen subjects had spiral CT thorax documented pulmonary embolism. For the whole group of 687 patients, the age ranged from 17 to 100, male to female ratio was 1 : 1.24. The commonest risk factors were malignancy (45%) followed by acute infections (24%), acute respiratory failure (16%), congestive heart failure (14%), and stroke (7%). The majority of the subjects (98%) did not receive antithrombotic prophylaxis during the current or previous admissions. There were no significant difference in age range sex ratio, and distribution of risk factors among the patients who developed DVT during hospitalization and those admitted with DVT. The calculated incidence of proximal DVT among medical patients hospitalized for at least two days were 1.8%, 2% and 1.7% for the year 2005, 2006 and 2007, respectively. The incidence was 1.1% for 2008 (*P* < .001). The incidence of pulmonary embolism for 2005, 2006, 2007, and 2008 were 4, 4, 5, and 4 cases, respectively. The incidence could be an underestimate because spiral CT were usually performed on subjects with severe dyspnea or hypoxia in the absence of heart failure or lung diseases. The demographic profiles of patients who developed proximal DVT from 2005–2008 were similar (Table 1).

## 4. Discussion

A majority of hospitalized patients have risk factors for deep vein thrombosis and pulmonary embolism, and hospitalization for medical illness is associated with an eight-fold-increased relative risk of VTE [13]. Fatal pulmonary embolism accounts for 7 to 10% of all hospital-related deaths, and may account for death in up to 1 in 200 medically ill hospitalized patients [14]. Randomized trials have demonstrated the value of prophylaxis for VTE in acutely ill medical patients. The MEDENOX [6], PREVENT [7], and ARTEMIS [8] studies showed statistically significant reductions in VTE versus. placebo by treatment with enoxaparin, dalteparin, or fondaparinux, respectively. There was little data about the incidence of DVT among Chinese patients admitted to a medical ward. In the past, it was widely believed that the incidence of DVT was much lower among the Chinese population than Caucasians, and prophylaxis was generally considered not necessary. 

Our study showed an annual proximal DVT incidence of 1.8–2% among Chinese patients admitted to the medical wards for the period 2005–07. For patients with risk factors such as malignancy, acute sepsis, acute respiratory failure or congestive heart failure, there was a 2–4-fold-increase in risk with incidence of 3.8–6.3% without prophylaxis [2]. Since 2008, there was increasing awareness that Chinese patients were not immune to the development of DVY and pulmonary embolism and the risk could be comparable to Western population. More and more physicians were willing prescribed antithrombotic therapy to hospitalized Chinese patients with risk factors. Our data showed that there was a significant decrease in the incidence of proximal DVT among medical patients in 2008 when compared to the previous years (1.1% versus 1.8–2%, *P* < .001). The decrease could be due to the increasing use of antithrombotic therapy as there was no significant difference in patient demographics and distribution of risk factors between 2005–7 and 2008. 

There are several limitations in our study. It was a retrospective study which probably would underestimate the true incidence of DVT. Many asymptomatic DVT would be missed. The true incidence of DVT among hospitalized Chinese patients without prophylaxis is uncertain. To obtain such data, one needs to perform Doppler ultrasound study prospectively on medical patients with various risk factors and not on any antithrombotic prophylaxis. Secondly, DVT prophylaxis was given at the discretion of the physicians rather than according to protocols or standardized practice. Therefore, some high-risk subjects would not receive any prophylaxis and vice versa. These would under estimate the benefits of DVT prophylaxis among Chinese patients.

Thirdly, Doppler ultrasound study was performed only in clinically suspected DVT cases. This may underestimate the incidence of DVT in patients receiving prophylaxis. However, the same bias would apply to patients not receiving prophylaxis.

In order to address the above limitations, we are now conducting a prospective study to assess the effectiveness of DVT prophylaxis in acutely ill medical patients admitted with respiratory failures, congestive heart failures or severe sepsis. So far none of the 107 recruited subjects had developed proximal DVT during their hospital stay or within 3 months of discharge. 

In conclusion, despite some limitations, our data did suggest that proximal DVT was substantial in Chinese medical patients and DVT prophylaxis might reduce such risk.

## Figures and Tables

**Table 1:** 

Year	2005	2006	2007	2008	2008 versus 2005–7
Proximal DVT incidence (cases detected)	1.8% (182)	2.0% (204)	1.7% (173)	1.1% (128)	*P* < .001
Age range	20–99	17–83	18–100	18–98	NS
Male : female ratio	1 : 1.2	1 : 1.36	1 : 1	1 : 1.4	NS

## References

[1] Vaitkus PT, Leizorovicz A, Cohen AT, Turpie AGG, Olsson CG, Goldhaber SZ (2005). Mortality rates and risk factors for asymptomatic deep vein thrombosis in medical patients. *Thrombosis and Haemostasis*.

[2] Barba R, Zapatero A, Losa JE (2010). Venous thromboembolism in acuteky ill hospitalized medical patients. *Thrombosis Research*.

[3] Alikhan R, Cohen AT, Combe S (2004). Risk factors for venous thromboembolism in hospitalized patients with acute medical illness. *Archives of Internal Medicine*.

[4] Cheng Y, Wong R, Lam J, Lau FY, Cheng G (2004). Estimated risk of deep venous thrombosis among Chinese patients admitted to general medical wards. *Journal of Thrombosis and Haemostasis*.

[5] Leung V, Leung V, Lui W, Chan T, Wong RSM, Cheng G (2006). Incidence of deep vein thrombosis in hospitalized Chinese medical patients is similar to that in western populations. *Thrombosis Research*.

[6] Heit JA, Silverstein MD, Mohr DN, Petterson TM, O’Fallon WM, Melton LJ (2000). Risk factors for deep vein thrombosis and pulmonary embolism: a population-based case-control study. *Archives of Internal Medicine*.

[7] Heit JA, Michael O’Fallon W, Petterson TM (2002). Relative impact of risk factors for deep vein thrombosis and pulmonary embolism: a population-based study. *Archives of Internal Medicine*.

[8] Samama MM, Cohen AT, Darmon JY (1999). A comparison of enoxaparin with placebo for the prevention of venous thromboembolism in acutely ill medical patients. *New England Journal of Medicine*.

[9] Leizorovicz A, Turpie AGG, Cohen AT, Dhillon KS, Angchaisuksiri P, Wang CJ (2004). Epidemiology of post-operative venous thromboembolism in Asian countries. *International Journal of Angiology*.

[10] Piovella F, Wang CJ, Lu H (2005). Deep-vein thrombosis rates after major orthopedic surgery in Asia. An epidemiological study based on postoperative screening with centrally adjudicated bilateral venography. *Journal of Thrombosis and Haemostasis*.

[11] Leizorovicz A, Cohen AT, Turpie AGG, Olsson CG, Vaitkus PT, Goldhaber SZ (2004). Randomized, placebo-controlled trial of dalteparin for the prevention of venous thromboembolism in acutely ill medical patients. *Circulation*.

[12] Cohen AT, Davidson BL, Gallus AS (2006). Efficacy and safety of fondaparinux for the prevention of venous thromboembolism in older acute medical patients: randomised placebo controlled trial. *British Medical Journal*.

[13] Goldhaber SZ, Dunn K, MacDougall RC (2000). New onset of venous thromboembolism among hospitalized patients at Brigham and Women’s Hospital is caused more often by prophylaxis failure than by withholding treatment. *Chest*.

[14] Pendleton R, Wheeler M, Rodgers G (2005). Venous thromboembolism prevention in the acutely III medical patient: a review of the literature and focus on special patient populations. *American Journal of Hematology*.

